# Clinical study protocol for a low-interventional study in intermediate age-related macular degeneration developing novel clinical endpoints for interventional clinical trials with a regulatory and patient access intention—MACUSTAR

**DOI:** 10.1186/s13063-020-04595-6

**Published:** 2020-07-18

**Authors:** Jan H. Terheyden, Frank G. Holz, Steffen Schmitz-Valckenberg, Anna Lüning, Matthias Schmid, Gary S. Rubin, Hannah Dunbar, Adnan Tufail, David P. Crabb, Alison Binns, Clara I. Sánchez, Carel Hoyng, Philippe Margaron, Nadia Zakaria, Mary Durbin, Ulrich Luhmann, Parisa Zamiri, José Cunha-Vaz, Cecília Martinho, Sergio Leal, Robert P. Finger, on behalf of the MACUSTAR consortium, P. Basile, C. Behning, M. Berger, A. Binns, M. Böttger, C. Bouchet, J. E. Brazier, T. Butt, C. Carapezzi, J. Carlton, A. Charil, R. Coimbra, S. Nunes, D. Crabb, J. Cunha-Vaz, H. Dunbar, M. Durbin, R. Finger, F. Holz, C. Hoyng, J. Krätzschmar, S. Leal, U. Luhmann, A. Lüning, Ph. Margaron, C. Martinho, B. Melício, S. Mohand-Saïd, D. Rowen, G. S. Rubin, J. Sahel, C. I. Sánchez, D. Sanches Fernandes, M. Schmid, S. Schmitz-Valckenberg, A. Skelly, L. Stöhr, D. Taylor, J. Terheyden, A. Tufail, L. Vieweg, L. Wintergerst, C. Wojek, N. Zakaria, P. Zamiri

**Affiliations:** 1 Department of Ophthalmology, University Hospital Bonn, Ernst-Abbe-Str. 2, 53127 Bonn, Germany; 2 Institute for Medical Biometry, Informatics and Epidemiology, Medical Faculty, University of Bonn, Bonn, Germany; 3 UCL Institute of Ophthalmology, University College London, London, UK; 4 Moorfields Eye Hospital, London, UK; 5 Division of Optometry and Visual Science, School of Health Sciences, City, University of London, London, UK; 6 Radboud University Medical Center, Nijmegen, Netherlands; 7 Novartis Pharma AG, Basel, Switzerland; 8 Carl Zeiss Meditec AG, Dublin, CA USA; 9 Roche Pharmaceutical Research and Early Development, Translational Medicine Ophthalmology, Roche Pharma Research and Early Development, Roche Innovation Center Basel, Basel, Switzerland; 10 Association for Innovation and Biomedical Research on Light and Image, Coimbra, Portugal; 11 Bayer AG, Berlin, Germany

**Keywords:** Intermediate age-related macular degeneration, Clinical study protocol, Disease progression, Clinical endpoint

## Abstract

**Background:**

There is an unmet need for treatment options in intermediate age-related macular degeneration (iAMD). However, for any new interventions to be tested in clinical trials, novel currently unavailable clinical endpoints need to be developed. Thus, the MACUSTAR study aims to develop and evaluate functional, structural, and patient-reported candidate endpoints for use in future iAMD trials.

**Methods:**

The protocol describes a low-interventional clinical multicenter study employing a novel two-part design. The cross-sectional part (total duration, 1 month) and the longitudinal part (total duration, 36 months) include participants with iAMD and control groups with early/late/no AMD. The cross-sectional part’s primary objective is a technical evaluation of functional, structural, and patient-reported candidate outcomes. The longitudinal part’s primary objective is to assess the prognostic power of changes in functional, structural, and patient-reported outcomes for progression from iAMD to late AMD. All data will be used to support a biomarker qualification procedure by regulatory authorities.

**Discussion:**

The MACUSTAR study characterizes and evaluates much needed novel functional, structural, and patient-reported endpoints for future clinical trials in iAMD and will improve our understanding of the natural history and prognostic markers of this condition.

**Trial registration:**

ClinicalTrials.gov NCT03349801. Registered on 22 November 2017

## Background

Age-related macular degeneration (AMD) is one of the main causes of irreversible severe visual loss in high-income countries, characterized by typical morphologic and functional changes of the central outer retina [1, 2]. The disease progresses slowly from early and intermediate AMD (iAMD) with drusen as a key finding, possible retinal pigmentary changes and none to moderate visual symptoms to late AMD with damage of the retinal pigment epithelium, atrophy or neovascularization and irreversible severe visual loss [3]. The most common form of late AMD is neovascular AMD (nAMD) which leads to legal blindness within on average 2 years [4]. After the introduction of vascular endothelial growth factor inhibitors, the outcome has significantly improved for these patients [5]. AMD currently affects more than 15 million individuals in the European Union (EU) alone and more than 190 million globally [2, 6–9]. With population aging, AMD prevalence is expected to increase considerably over the next decades [2, 6].

Considering this background, novel treatment options which prevent or slow down disease progression from iAMD to late AMD and biomarkers that allow precise identification of individuals at risk of progression are urgently needed [10]. Currently, no validated clinical endpoints accepted by regulatory agencies, payers, and health technology assessment (HTA) bodies are available. Best-corrected visual acuity (BCVA) at high contrast and high luminance is accepted as the only primary study endpoint for neovascular AMD trials by authorities to date, but this marker does not detect the functional deficit in early or iAMD sensitively [11, 12]. Individuals affected by iAMD frequently report difficulties seeing in dim or changing light conditions as well as in low-contrast surroundings [13, 14]. Visual function tests designed to measure functional impairment under these circumstances may be able to characterize the visual deficit in individuals with early and iAMD and might allow for a more accurate prediction of the individual risk of disease progression as well as be more patient-relevant [15–17]. For these reasons, novel clinical endpoints for iAMD trials which are clinically meaningful and acceptable to regulatory agencies need to be developed and validated. Besides single outcome measures, combined parameters could function as candidate endpoints for future trials. The evaluation of different promising parameters not only from a functional point of view but from a structural and a patient-reported standpoint is a prerequisite to developing endpoints. Furthermore, the natural history of iAMD as well as risk factors associated with disease progression to late AMD needs to be better understood.

Overall, there is an urgent and unmet need for adequate tools which would allow for interventional trials to be conducted in early stages of AMD. The MACUSTAR study aims to fill this gap and develop and evaluate functional, structural, and patient-reported candidate endpoints for future use in iAMD trials.

## Methods/design

The MACUSTAR clinical study consists of two parts and combining a cross-sectional and a longitudinal component to achieve the study objectives (see below). In the cross-sectional part, four groups of participants with different stages of disease (targets no AMD/normal aging changes, *n* = 50; early AMD, *n* = 50; iAMD, *n* = 150; late AMD, *n* = 50) are planned to be included. The analysis of this part incorporates two visits: the baseline visit at day 0 and a short-term follow-up visit at day 14 (± 7 days). In the longitudinal part, a group of iAMD participants (target *n* = 600: *n* = 150 from the cross-sectional part, and *n* = 450 additional subjects) as well as the participants with early AMD (target *n* = 50, recruited in the cross-sectional part) are followed up for a 3-year period. The rationale of the study and the justification for the sample size have been described previously [18]. The MACUSTAR clinical study is conducted, analyzed, and reported according to current international laws and regulations and in compliance with regulatory requirements in all participating countries and the standards of good clinical practice according to the International Council for Harmonisation of Technical Requirements for Pharmaceuticals for Human Use. This includes the submission of study protocol and any amendments to the respective institutional review board/ethics committee. This publication refers to protocol version 5.0 as approved on 15 July 2019. Future modifications will be shared with the investigators, institutional ethics committees/review boards, and the scientific community as indicated.

### Study objectives

The main objective of the MACUSTAR study is to develop novel clinical endpoints for future clinical trials with a regulatory and patient access intention in patients with iAMD. Additional objectives are the characterization of visual impairment in iAMD and its progression and the identification of risk factors for progression to late AMD (Table 1).

**Table 1:** Objectives of the MACUSTAR study

	Cross-sectional part	Longitudinal part
**Primary objectives**	Technical evaluation of functional and structural candidate outcomes	Assessment of prognostic power of functional, structural, and patient-reported outcomes for progression from iAMD to late AMD
**Secondary objectives**	Correlation analyses between defined structural, functional, and patient-reported outcome measures; Evaluation of discriminative ability of outcome measures (with respect to AMD stages and participants without AMD); Implementation of a database for no AMD, early AMD, iAMD, and late AMD	Correlation analyses between defined structural, functional, and patient-reported outcome measures over time; Evaluation of the natural history of iAMD biomarkers
**Exploratory objectives**	Hypothesis-free testing of associations/correlations	Identification of sub-stages of iAMD that relate to progression to late AMD; Assessment of the association of single and combined endpoints to the final status of patients (progressed vs. not progressed); Hypothesis-free testing of associations/correlations

### Study outcomes

The MACUSTAR study collects data in relation to functional, structural, and patient-reported outcomes, including best-corrected visual acuity, mean and point-wise retinal sensitivities on scotopic/mesopic fundus-controlled perimetry, low-luminance visual acuity (LLVA), low-luminance deficit, Moorfields acuity chart visual acuity, absolute threshold and rod intercept time of dark adaptation, time to complete mobility course time, walking speed and error rate, reading speed in words per minute and number of words read correctly, central subfield retinal thickness, total retinal volume, presence of refractile deposits, intraretinal cystoid spaces, choroidal neovascularization, maximum drusen size, presence of pigment epithelium detachment, pigmentary abnormalities, reticular drusen, focal increased autofluorescence signal, vitelliform material, exsudation, geographic atrophy, and patient-reported low-luminance functioning (Vision Impairment in Low Luminance questionnaire). Besides this, demographic data (year of birth, weight, height, gender, race) and medical and ophthalmic history, concomitant medications, adverse events, and prior non-ocular and ocular treatments are documented. Information on the inclusion and exclusion and results of the physical examination (blood pressure, heart rate) and ophthalmologic examination (external examination; screening for eyelid/pupil responsiveness; slit-lamp examination of the cornea, lens, and iris; indirect ophthalmoscopy; assessment of intraocular pressure; refraction; dilated fundus examination of the optic nerve; peripheral retina and retinal vasculature) are also recorded.

### Design of the study

The MACUSTAR clinical study is a low-interventional clinical multicenter study (i.e., no therapeutic intervention, predefined set of subject assessments) to develop novel candidate clinical endpoints for iAMD. The recruitment period was planned to be 21 months. The participants are recruited at 20 clinical sites from seven European countries (Denmark, France, Germany, Italy, Netherlands, Portugal, UK). The cross-sectional part (total duration, 1 month) and the longitudinal part (total duration, 36 months) plan to enroll 750 participants in total. Three hundred individuals are planned be included in the cross-sectional part (no AMD, *n* = 50; early AMD, *n* = 50; late AMD, *n* = 50; iAMD, *n* = 150), and 650 participants are planned to be included in the longitudinal part (iAMD, *n* = 600; early AMD, *n* = 50). The study consists of three phases: screening, inclusion, and follow-up phases.

#### Screening phase

The screening period (maximum duration, 4 weeks) starts with the first study visit (V1). No treatment is allowed during this phase except for late AMD subjects and iAMD subjects included in the longitudinal part of the study (unilateral iAMD). Participants are recruited at the local level. Potential participants are recruited, e.g., at consultations and from pre-existing study participant databases. The participants who sign the informed consent form (ICF) perform the study procedures. The investigator must ensure that each subject is fully informed about the nature and objective of the study and possible risks associated with participation. Imaging data obtained are transferred to the central reading center (GRADE Reading Center, Bonn, Germany). Participants’ eligibility is evaluated by the local investigator based on the inclusion and exclusion criteria (see below) and on the disease stage of both eyes, as based on the clinical classification of AMD according to the Beckman classification [19]. Eligible participants are assigned to one of the four groups included in the study: no AMD, early AMD, iAMD, or late AMD. If both eyes are eligible for the study based on inclusion criteria, the eye with better visual acuity is selected as the study eye. In cases in which both eyes have the same visual acuity, the study eye is selected by the investigator. Participants’ eligibility with regard to the image-related inclusion/exclusion criteria and slot availability for the respective group is finally confirmed by the central reading center within 48 h.

#### Inclusion and exclusion criteria

The following criteria determine eligibility for the MACUSTAR study:

General inclusion criteria

1. Male and female participants.
2. Aged 55–85 years at baseline.
3. Able and willing to provide written informed consent and to comply with the study protocol visits and assessments. In case the individual is physically incapable of signing the ICF, this can be provided by a legally acceptable representative of the participant or impartial witness.

Specific inclusion criteria for the iAMD group:

1. The study eye must have iAMD (defined by large drusen > 125 μm and/or any AMD pigmentary abnormalities that are definite hyper- or hypopigmentary abnormalities associated with medium or large drusen but not associated with other known disease entities) and
2. Individuals to be included in the
  - *Cross-sectional part*: The fellow eye must have iAMD and/or, in addition, extrafoveal geographic atrophy (GA, no atrophy within the central ETDRS subfield); maximum total GA size is 1.25 mm^2^.
  - *Longitudinal part*: The fellow eye must have AMD (early AMD, iAMD, or late AMD—any stage of AMD is allowed).
3. Early Treatment Diabetic Retinopathy Study (ETDRS) letter chart BCVA in the study eye not worse than 72 letters (approximately 20/40 Snellen visual acuity equivalent).
4. All general inclusion criteria.

Specific inclusion criteria for the late AMD group:

1. Individuals with bilateral GA, bilateral nAMD, or nAMD in one eye and GA in the other.
  GA is defined as a retinal area at minimum size of 0.1 mm^2^ that shows a severely decreased fundus autofluorescence signal and correlates to outer nuclear layer loss and an enhanced signal of the choroid on spectral domain-optical coherence tomography (SD-OCT). In addition, it needs to be of limited size and show AMD typical changes on color fundus photography (CFP) such as depigmentation, hyperpigmentation, and crystalline deposits if hemorrhages or exudates are absent.
  Choroidal neovascularization (CNV) is defined based on CFP, SD-OCT, and/or fluorescein angiography (FA, optional). At least two of the following signs must be present within a radius of 3000 μm of the fovea to confirm CNV: (1) serous detachment of the sensory retina, (2) subretinal/retinal hemorrhage, (3) pigment epithelial detachment (excl. drusenoid pigment epithelial detachment), (4) fibrous tissue, (5) hard exudates, and (6) disciform scar. FA-confirmed CNV (with corroborating evidence on OCT) is sufficient without clinical signs of CNV.
2. BCVA between 20/80 and 20/200 in the study eye.
3. All general inclusion criteria.

Specific inclusion criteria for the early AMD group:

1. Individuals with medium drusen > 63 μm and ≤ 125 μm and no AMD pigmentary abnormalities in both eyes and no signs of intermediate or late AMD.
2. All general inclusion criteria.

Specific inclusion criteria for the no AMD group:

1. No signs of early, intermediate, or late AMD in both eyes.
2. All general inclusion criteria.

General exclusion criteria:

1. Media opacity or eye movement disorder (nystagmus) that interferes with retinal imaging data quality in the opinion of the investigator.
2. Severe ptosis, extraocular motility restriction, or head tremor preventing adequate fundus visualization in the opinion of the investigator.
3. Any signs of nAMD or GA (does not apply to the late AMD group and to fellow eyes within the intermediate AMD group for individuals to be included in the longitudinal part only).
4. Any concurrent intraocular condition in the study eye (e.g., glaucoma or cataract) that in the opinion of the investigator would either require surgical intervention during the study to prevent or treat visual loss that might result from that condition or affect interpretation of study results.
5. Severe non-proliferative diabetic retinopathy, or proliferative diabetic retinopathy.
6. Any diabetic macular edema or macular disease.
7. Ocular disorders in the study eye (i.e., pre-retinal membrane) at the time of enrolment that may confound interpretation of study results and compromise visual acuity.
8. Diagnosis of uncontrolled glaucoma with intraocular pressure of > 30 mmHg (despite current pharmacological or non-pharmacological treatment).
9. Known systemic illness which in the opinion of the investigator will prevent from actively participating in the study.
10. Concomitant treatment for AMD in either eye (concomitant use of vitamins/supplements is not excluded; does not apply to the late AMD group and to fellow eyes within the intermediate AMD group for individuals to be included in the longitudinal part only).
11. Any periocular or intravitreal injections (IVT) in either eye (does not apply to the late AMD group and to fellow eyes within the intermediate AMD group for individuals to be included in the longitudinal part only).
12. Participation in any other interventional trial.
13. Obvious retinal changes due to causes other than AMD (e.g., evidenced by an existing diagnosis of monogenetic macular dystrophies, Stargardt disease, cone rod dystrophy, or toxic maculopathies).
14. Any history of allergies to fluorescein.
15. Cognitive impaired individuals, illiterate, and individuals who do not speak the national language.

Specific exclusion criteria for the iAMD group:

1. Any GA in the study eye.
2. Any extrafoveal GA larger than 1.25 mm^2^ (as defined above) in the fellow eye (only applies to individuals to be included in the cross-sectional part).
3. All general exclusion criteria.

Specific exclusion criteria for the late AMD group:

1. All general exclusion criteria only.

Specific exclusion criteria for the early AMD group:

1. Intermediate or late AMD (following Beckman classification) in any eye.
2. All general exclusion criteria.

Specific exclusion criteria for the no AMD group:

1. Early to late AMD (following Beckman classification) in any eye.
2. All general exclusion criteria.

#### Inclusion phase

The inclusion phase contains the baseline visit (V2). If an individual is eligible after screening and participant slots are available for the respective group, V2 is scheduled within 30 days from the screening visit (V1). At the baseline visit, the clinical site confirms the inclusion/exclusion criteria to ensure that the individual remains eligible. Participants are informed about the planned number of follow-up visits and the duration of their participation in the study (Table 2).

**Table 2:** Visits to be performed according to the AMD group

Study phase	Screening	Baseline	Follow-up						
Visit number	1	2	3	4	5	6	7	8	9
Visit day/month	− 28D	0	14D	6M	12M	18M	24M	30M	36M
No AMD	X	X	X						
Early AMD	X	X	X		X		X		X
Late AMD	X	X	X						
Intermediate AMD	X	X	X*	X	X	X	X	X	X

*This visit is only performed by subjects included on the cross-sectional part

#### Follow-up phase

The follow-up phase comprises visit 3 (day 14) to visit 9 (month 36). Patients who progress from early or iAMD to late AMD during follow-up remain in the study for the whole follow-up period and are examined according to the Clinical Study Protocol. The respective patients can be treated according to the standard of care if deemed necessary by the treating physician. If a participant prematurely terminates the study, the investigator should attempt to complete the assessments of visit 9 (discharge visit). These data are planned to be included in the analysis unless consent is withdrawn.

Unscheduled visits may be necessary during the study as part of the assessment of adverse events or other safety concerns. The investigator should consider performing only the necessary study procedures, on a case-by-case basis. The acquired imaging data, if any, should be transferred to the central reading center, and an unscheduled visit must be completed on the electronic case report form (eCRF).

### Study procedures

The assessments have been structured in a way that allows the study participants to perform less and more demanding tests in an order that reduces signs of fatigue for the participants and allows the examiner to obtain the data in an efficient way. Examinations requiring pupil dilation are performed in the second part of the study visit since mainly the chart-based functional tests have to be administered to patients prior to pupil dilation. In the screening visit, obtaining consent from the participant is the mandatory first step prior to any study examinations. The individual clinical sites are allowed to adapt the following visit schedule recommendation developed for MACUSTAR to the local settings. Staff training and, for some procedures, certification are required before data are acquired.

Blood collection and chart-based function tests (refraction and best-corrected visual acuity according to the ETDRS protocol, in sitting position at an initial testing distance of 4 m, Moorfields acuity test, low-luminance visual acuity, contrast sensitivity) are recommended to precede the assessment of reading speed. For sites equipped for navigational testing, the respective course is performed on a separate visit within 21 days. The interview-based administration of the VILL and EQ5D-5 L questionnaires can be performed after application of dilating eye drops for the following examinations. Fundus imaging including OCT, CFP, and scanning laser ophthalmoscopy (see study schedule, Table 3) are recommended to be performed next, followed by two examinations that require prior dark adaptation (5 min for mesopic fundus-controlled perimetry [also called microperimetry], additional 30 min for scotopic testing). Details on these examinations performed have been described previously [18]. All images are acquired by certified staff and evaluated centrally by trained personnel at the central reading center. Staff certifications are based on respective standard operating procedures and contain the upload of images and/or passing a written examination on the respective procedures. Lastly, the ophthalmological examination may complete the study visit. The overall duration of a MACUSTAR visit was tested to be about 4 h which participants reported to be acceptable.

**Table 3:** Study schedule of MACUSTAR

Study phase	Screening	Baseline	Follow-up						
Visit number	1	2	3*	4	5	6	7	8	9
Visit day/month	− 28D	0	14D	6M	12M	18 M	24M	30M	36M
Allowed window	Up to 4-week interval		± 7D	± 1M	± 1M	± 1M	± 1M	± 1M	± 1M
**Procedures**									
Informed consent (clinical study)	X								
Informed consent (genetics/biosamples)	X								
History (medical + ocular)	X								
Inclusion/exclusion criteria	X	(X)^1^							
Demographics	X								
Consultation with physician	X	X	X	X	X	X	X	X	X
VILL questionnaire		X	X	X	X	X	X	X	X
EQ-5D-5L questionnaire		X			X		X		X
Funduscopy	OU	OU	OU	OU	OU	OU	OU	OU	OU
Slit-lamp examination	OU	OU	OU	OU	OU	OU	OU	OU	OU
Intraocular pressure	OU	OU	OU	OU	OU	OU	OU	OU	OU
Refraction	OU	OU		OU	OU	OU	OU	OU	OU
BCVA	OU	OU	OU	OU	OU	OU	OU	OU	OU
LLVA		OU	OU	OU	OU	OU	OU	OU	OU
Moorfields Acuity Test (MAT)		OU	OU	OU	OU	OU	OU	OU	OU
Contrast sensitivity (Pelli Robson)		OU	OU	OU	OU	OU	OU	OU	OU
Reading test (International Reading Speed Texts)^3^		X	X	X	X	X	X	X	X
Fundus-controlled perimetry (scotopic + mesopic)^7^		SE	SE	SE	SE	SE	SE	SE	SE
Absolute threshold (dark adaptation)^7^		SE	SE	SE	SE	SE	SE	SE	SE
Spectralis imaging (cSLO, fundus autofluorescence, SD-OCT)^7^	OU		OU	SE	SE	OU	SE	SE	OU
Cirrus OCT imaging^7^	OU		OU	SE	SE	OU	SE	SE	OU
Color fundus photography^7^	OU		OU	SE	SE	OU	SE	SE	OU
Fluorescein angiography^2,7^	(OU)	(OU)	(OU)	(OU)	(OU)	(OU)	(OU)	(OU)	(OU)
OCT-angiography^3,7^		OU	SE	SE	SE	OU	SE	SE	OU
Swept-source OCT^3,7^		OU	SE	SE	SE	OU	SE	SE	OU
Adaptive optics imaging^3^		OU	SE	SE	SE	OU	SE	SE	OU
Quantitative fundus autofluorescence^3,7^		OU	SE	SE	SE	OU	SE	SE	OU
**Additional assessments**									
Navigation performance^4^		X							
Blood sampling—Biobanking^5^		X			X		X		X
Blood sampling—genetics		X^6^							
Concomitant medications		X		X	X	X	X	X	X
Adverse events		X	X	X	X	X	X	X	X

*D* days, *M* Months, *OU* both eyes (eyes are tested separately), *SE* study eye. *This visit is only performed by subjects on the cross-sectional part. ^1^Check for eligibility based on CRC assessment. ^2^To be done at the study site when there is suspicion of conversion to CNV. ^3^At equipped sites only. ^4^At selected sites only. ^5^Plasma, serum, and DNA are biobanked for future analyses. ^6^Blood collection for Genetics should occur at baseline visit, but it can occur at any time during the subject follow-up. ^7^Images to be sent to the central reading center

### Study completion

Participants have achieved study completion if the following visits are completed successfully: V3 for individuals participating in the cross-sectional part only (participants with no AMD and late AMD) and V9 for subjects participating in the longitudinal part (participants with iAMD and early AMD). The end of the clinical study corresponds to the last visit of the last participant. The study can be prematurely terminated at any time by the sponsor for any sufficient reason. Overall, patients with iAMD are at risk of progression to late AMD. Providing state-of-the-art high-resolution retinal imaging and monitoring progression, which are thus potentially picked up early, will create a large incentive for the patients to remain in the study. We will also provide a capped travel reimbursement if requested by the patient.

### Safety assessments

The MACUSTAR study is a low-interventional study, and it does not investigate any therapeutic interventions. All devices used are already marketed in the study countries or are market-ready. As almost all assessments and procedures (except for FA and blood collection) are non-invasive, most adverse events may be due to the natural progression of the underlying disease or occurrence of concomitant diseases. All adverse events (defined as any unfavorable and unintended sign, symptom or disease temporally associated with the use of any study procedure, whether or not considered related to the study procedure) and serious adverse events (defined as any untoward medical occurrence or effect that results in death, is life threatening, requires hospitalization or prolongation of existing hospitalization, results in persistent or significant disability or incapacity, is a congenital anomaly or birth defect or is an important medical event) are collected in the eCRF. The serious adverse events are reported in a serious adverse event report form. The conversion to late AMD does not fulfill the criteria of a serious adverse event according to the study criteria.

### Data management and quality assurance

All clinical data are entered by the investigators or designated staff into the eCRF. Clinical staff is given access to eCRF after training on a platform in advance. Imaging data will be exchanged between clinical sites and the central reading center through an internet portal and stored on a secure server. All data are stored in pseudonymized form.

Monitoring happens throughout the study with clinical monitors from the European clinical research infrastructure network visiting the clinical site for site initiation visit followed by regular 6 monthly visits throughout the study to ensure compliance with the study protocol as well as regulatory requirements and good clinical practice (according to the International Council for Harmonisation of Technical Requirements for Pharmaceuticals for Human Use). Monitors and the data management team review the eCRF for completeness and accuracy and instruct clinical site personnel to make any required corrections or additions. During monitoring visits, monitors will ensure that the data included in the eCRF is supported by data in the source documents. Queries are sent to the clinical site as applicable. Designated clinical site staff is required to respond to the queries and make the necessary changes to the data. Data management is performed using automatic data validation requests within the eCRF, supplementary periodic validation checks performed by the data management team. Missing data detected and queries raised on this basis are regularly communicated to the clinical sites and the monitors. Finally, manual data validation checks are implemented by the data management team as needed.

Missing values are regularly analyzed during the course of the study. Missing values are replaced using multiple imputation with 10 imputed data sets wherever possible. Variables with more than one third missing values in any of the visits will be checked for visit-wise exclusion from statistical analysis. If a variable contains less than or equal to 3% missing values per visit, these missing values will be imputed using univariate mean, median, or mode imputation. Complete-case analyses will be carried out in order to investigate the sensitivity of results with regard to multiple imputation procedures.

### Dissemination of results

Upon study completion and final study report, the results of this study will be submitted for publication in order to share the achieved scientific results with peers at local and international journals/congresses. Authorship rules follow the guidelines of the international committee of medical journal editors. The MACUSTAR data access and publication committees coordinate these efforts. Relevant results will be shared with patient organizations and the general public in press releases.

### Regulator interaction

A joint scientific advice procedure with the European Medicines Agency (EMA), Food and Drug Administration (FDA), and Health Technology Assessment (HTA) bodies (in this case NICE, UK) on the proposed MACUSTAR clinical study design and selected outcome measures was initiated by the MACUSTAR consortium in 2016 prior to study start. The regulators supported the approach of the study to develop and validate functional and structural clinical endpoints with patient-reported outcome measures as secondary endpoints for future clinical trials on iAMD. Also, they advised to share relevant study results supporting endpoint validation with all regulatory bodies. Following this, EMA published a letter of support for the MACUSTAR clinical study (available at www.ema.europa.eu). Further scientific advice procedures are planned once clinical study data become available.

## Discussion

Considering the expected increase in affected individuals with AMD due to population aging as well as the considerable adverse impact of AMD on the affected person, their families, and society at large, we need to urgently pave the way for the development of interventions to prevent, stop, or delay the disease in its early stages. The MACUSTAR study will deliver necessary tools to conduct these much needed clinical trials and enable future development of early and iAMD interventions. An ongoing dialogue with regulators will ensure that regulator expectations and feedback are met and increase the likelihood of developed outcomes being accepted as clinical endpoints by regulators.

Multiple functional tests included in the MACUSTAR study will be able to comprehensively characterize the functional deficit of AMD patients, mainly present at low light levels and in low contrast situations [15, 20, 21]. The functional testing will be complemented by innovative multimodal imaging modalities and patient-reported outcome measures optimized for the specific functional impairment in early AMD stages. Out of the functional testing employed, fundus-controlled perimetry [22, 23] is a promising functional candidate endpoint in iAMD as it could characterize the localized functional deficit in AMD well in previous studies [11, 13, 24–28]. The MACUSTAR study implements its mesopic and scotopic variants to simulate conditions under which persons with early and iAMD are expected to experience functional deficits. For example, visual impairment after dark adaptation has been shown to be related to retinal structural changes in individuals with iAMD [25]. Other functional candidate endpoints include dark adaptometry and chart-based tests. An impaired process of dark adaptation in healthy individuals predicted AMD onset over 3 years [29], and there is evidence of its association with disease progression [30]. Chart-based tests are easy and rapid to administer and have shown associations with AMD progression [31–33]. For this purpose, different types including low-luminance visual acuity and contrast sensitivity are used as candidate functional endpoints in the MACUSTAR study. However, no long-term data in large cohorts exist for these functional parameters as of yet.

Different to functional outcome measure, structural outcome measures allow for a participant-independent, objective assessment of disease stage and progression. AMD is characterized by a number of retinal biomarkers which are captured in MACUSTAR using high-resolution, non-invasive retinal imaging. These current imaging modalities are known to be more sensitive in the detection of advanced AMD and allow for the evaluation of a wider variety of structural endpoints [34–36]. Implementing state-of-the-art, multimodal retinal imaging technologies, large volumes of data are created. Automated and semi-automated quantification of imaging biomarkers have shown promising results in the detection and quantification of retinal AMD biomarkers [37–39]. In the MACUSTAR study, we are able to compare such new approaches to the current gold standard of manual grading by a central reading center as well as assess combined structural and functional outcome measures.

Endpoints combining functional and structural data have become increasingly relevant for studies of slowly progressing diseases such as AMD or glaucoma. In glaucoma clinical research, not only the slow progression of the disease but also the considerable noise in perimetry-based functional endpoints historically necessitated very large sample sizes [40]. Adding a structural dimension to a functional endpoint has shown to improve predictive accuracy for short-term data, increase the statistical power of tests, and therefore allow for reduced sample sizes in clinical trials [41, 42]. We will explore combined functional and structural endpoints in iAMD following this prescription from glaucoma clinical research.

Patient-reported outcomes (PRO) are increasingly important in clinical research. As many of the available vision-related PRO instruments either do not capture the specific visual impairment in early AMD stages or have not been developed with regulatory requirements in mind, we use a newly and specifically developed PRO, the Vision Impairment in Low Luminance (VILL) questionnaire in the MACUSTAR study. The PRO will aid in determining patient relevance of changes in structure and function.

Surrogate endpoints are required in many slowly progressive diseases or when events of interest occur rarely or are difficult to capture [43]. They have been widely used in clinical trials in ophthalmology with, for instance, intraocular pressure being one of the most common endpoints in glaucoma trials [44, 45]. In AMD clinical trials, only high-luminance, high-contrast best-corrected visual acuity has been available as a regulator accepted primary efficacy outcome measure for the approval of new therapeutics in ophthalmology [45], an outcome measure inapplicable to earlier AMD stages. Following the 2016 Endpoints Workshop on Age-Related Macular Degeneration and Inherited Retinal Diseases organized by the National Eye Institute (NEI, National Institute of Health, USA) and the United States Food and Drug Administration (FDA), it was noted that structural candidate endpoints require evident strong correlations with functional biomarkers in order to become accepted trial endpoints [12]. Patient-reported outcomes were proposed as meaningful secondary outcome measures for future trials in AMD by regulatory agencies and therefore should also be considered [45]. Despite available data on functional, structural, and patient-reported outcome measures in iAMD, surrogate biomarkers require careful validation to avoid potential harm as seen by regulators [46, 47]. According to the well-established Prentice criteria, the effect of a treatment is always mediated by true surrogate endpoints, which is a unidirectional process. The hard outcome measure must not be affected by the treatment in any parallel way [48, 49]. Applied to the development of clinical trial endpoints in AMD, candidate endpoints need to be understood profoundly in both a longitudinal and a cross-sectional setting, which will be provided in the MACUSTAR study.

With more than 190 million people currently affected by AMD globally [9], there is an urgent, yet unmet need for interventions to prevent, stop, or delay AMD and its progression. For this, however, we need to be able to conduct efficient clinical trials with appropriate clinical endpoints accepted by regulators. Considering this background, the MACUSTAR study will develop appropriate outcome measures and engage with regulators to establish clinical trial endpoints for future clinical trials in iAMD to fill this gap.

## Trial status

Current protocol version: 5.0 (15 July 2019); currently recruiting (since 01 March 2018).

## Supplementary information

**Additional file 1.** Procedure Manual. PROM Administration: VILL and EQ-5D-5L

**Additional file 2.** Procedure Manual. Retinal Imaging

**Additional file 3.** Model consent form.

## Data Availability

Not applicable
