# Supplementary material for: Clinical study protocol for a low-interventional study in intermediate age-related macular degeneration developing novel clinical endpoints for interventional clinical trials with a regulatory and patient access intention—MACUSTAR
Source: Trials. 2020 Jul 18;21:659. doi: 10.1186/s13063-020-04595-6 (PMC7368769; doi:10.1186/s13063-020-04595-6)
Supplement: Supplementary file 1 — Additional file 1. Procedure Manual. PROM Administration: VILL and EQ-5D-5L [file 13063_2020_4595_MOESM1_ESM.pdf]

Procedure Manual

PROM Administration: VILL and EQ-5D-5L

MACUSTAR Study

ECR-AMD-2017-13

Version 1.1

1 Introduction

This document describes the procedures to administer patient-reported outcome (PRO) questionnaires for the Clinical Study "Development of novel clinical endpoints for interventional clinical trials with a regulatory and patient access intention in patients with intermediate age-related macular degeneration (AMD) – MACUSTAR"(Protocol no ECR-AMD-2017-13) to ensure that a uniform procedure is followed by all clinical sites participating in the study, in order to obtain comparable and reliable data, as according to International Conference on Harmonization Good Clinical Practice (ICH-GCP) guidelines. This procedure will be performed to the study subjects according to the Clinical Study Protocol.

To be able to assess the impact on visual impairment on subjects' lives, the following PRO questionnaires will be used in the MACUSTAR clinical study:

- Vision Impairment in Low Luminance (VILL) questionnaire
- EQ-5D-5L questionnaire

Prior to administration of PRO questionnaires, each site must have the appropriate personnel trained for PRO questionnaire administration (see chapter3).

2 Study overview

An overview of the MACUSTAR study visits with PRO questionnaire administration scheduled can be found in the current version of the study protocol.

3 Training of study personnel

The principal investigator is responsible for ensuring that the appropriate personnel for administration of the PRO questionnaires is identified (see 'Delegation of Duties' documentation in the Investigators Site File) and trained. For training, all site personnel administrating the questionnaires have to read this manual before administering any questionnaires in the study. It has to be documented by the respective site personnel and the principal investigator that they have read and understood the instructions given in this manual on the Staff Training Log in the Investigator File section 1.

No formal certification is required for PRO questionnaire administration, i.e. no certificate will be issued.

Clinical sites are recommended to have a minimum of two staff members in the study team trained for this procedure.

4 PROM Administration

4.1 Introduction

Both questionnaires (VILL and EQ-5D-5L) will be administered at all clinical sites to all participants according to the study protocol. Both questionnaires will be subject self-administered. In case the subject requests help from the site personnel, the questionnaires can also be interviewer-administered.

Filling out the questionnaires or administering the questionnaires may take up to 30 minutes. This time may vary from subject to subject and from visit to visit. The subject should have sufficient time to complete the questionnaires. Specific instructions for the different questionnaires are given on the questionnaires themselves.

4.2 Preparation

The responsible site personnel shall

- Administer the VILL and EQ-5D-5L questionnaires in an area that is quiet and has adequate privacy and make the subject as comfortable as possible.
- Administer the questionnaires to the subjects in private. A family member or accompanying person may stay with the study subject if requested or clinically indicated.
- Re-assure the subjects that their answers are kept confidential and will not affect the study participation.
- Explain the procedure to the subject orally, e.g. that they will answer a series of questions and this is an important part of the MACUSTAR clinical study to obtain information on the impact of visual impairment on subjects' lives.
- Explain that always only **ONE** box should be ticked or crossed per question.
- Explain that the subject should **NOT** complete the header and administration mode section on the last page, both highlighted in grey. These sections have to be completed by the site personnel.
- Ensure that subjects (and site personnel) write on a firm writing surface with a **black pen**.
- Complete the header (fields highlighted in grey in VILL questionnaires) with the subject identification code ("313", 3 digits for the site number, 4 sequential digits for the subject number) and visit information. Personalized data (e.g. date of birth, name, etc.) should **NOT** be noted on the questionnaire.

- Please note that the 2 pages of the **EQ-5D-5L** questionnaire do not include a header section. **Add the subject identification code (see above), visit date and visit number by hand** on the top of each page. On the second page of the EQ-5D-5L questionnaire, add the **administration mode** (subject self-completed or interviewer-administered) **by hand**.

4.3 Administration of PRO questionnaires

All questionnaires shall be administered before any examination requiring pupil dilation to ensure that the subject is able to read the questions without reading problems caused by the pupillary dilation medication. In case the questionnaires are administered after pupil dilation, the questionnaires have to be interviewer-administered. In case the questionnaires are administered during the time the subject is dark adapting for microperimetry assessments, the responsible site personnel has to follow the instructions in chapter 4.4. A red LED light torch can be used by the site personnel to be able to read the questions in the dark. The responsible site personnel shall

- Make the subjects complete the questionnaires as outlined in the current version of the study protocol according to the specific instructions given on the questionnaire itself. Always follow the order described in the protocol and start with the VILL questionnaire followed by the EQ-5D-5L.
- Complete the questionnaires during the visit, don't hand out a copy to the subject to be completed afterwards, i.e. at home, to ensure that all the questions are answered without any influences or distractions.
- When the subject reads the questionnaire individually or along with the interviewer, the subject may use their glasses or visual aids if needed. The site personnel may also offer visual aids available at the site to the subject if needed.

4.4 During the PRO questionnaire administration

Both questionnaires are self-administered questionnaires and can be completed by the subjects themselves. If a subject is unable to self-administer the questionnaire (e.g. due to reading difficulty) the site personnel should offer to complete the questionnaire interviewer-administered. In case of interviewer-administered completion, the interviewer should proceed as follows:

- Always start with the VILL questionnaire followed by the EQ-5D-5L.
- Read the instructions on the questionnaires to the subject and ensure that the subject understands the instructions and is ready to start.

- Read each question and the respective answer choices slowly and mark the chosen answer for the subject. If needed, repeat questions and answer choices.
- Do not influence the subject to particular answers. If subjects are unsure how to answer a question, re-assure the subject that there are no right or wrong answers and ask them to answer to the best of their ability.
- If the subject changes their answer after one answer choice has been marked, cross out and initial the wrong answer, and mark the new one.
- Move the interview forward not to get stuck on particular questions.

4.5 Completing the PRO questionnaire administration

After the questionnaire has been completed, thank the subject for participation and check the following on the questionnaire:

- The header information corresponds to the subject identification code, visit day and schedule.
- All questionnaires were answered and all pages completed.
- In case of missing answers, the site personnel should **NOT** correct any omissions, errors, or discrepancies. If complete pages were not answered by the subject the site staff has to ask whether this was forgotten or whether the subject chose not to answer any questions on that page. If it was just forgotten, the subject should be handed the questionnaire back to answer all questions on this page.
- Complete the administration mode information on the last page (highlighted in grey in VILL questionnaire) and add id by hand on the second page of EQ-5D-5L questionnaire.
- The completed paper versions of the questionnaires are considered source documents. No photocopies should be made or used instead of the original forms.
- If any information regarding adverse events is reported by the subject during the questionnaire administration, ensure that this information is documented appropriately.
- Enter the subjects' responses from the questionnaire into the electronic case report form (eCRF).
- File the questionnaires in the subject folder at the applicable visit.

5 Frequently asked questions

The subjects may ask the site personnel several general questions regarding the administration procedure or have objections against completing the questionnaires. Some common examples of those questions and responses can be found below.

Instructions how to answer questions of understanding of several questions of the VILL questionnaire can be found in the next section.

Q: "I don't feel/cannot see or read very well. Do I have to complete the questionnaire?"

- Empathise with the subjects and stress the importance of completing the questionnaire.
- Remind the subject that all subjects must complete this questionnaire as part of the study procedures and that the data are important to assess the impact of visual impairment on subjects' lives.
- Offer the subject to complete the questionnaire interviewer administered if needed.

Q: "Can I take/complete the questionnaire (at) home?"

- Explain that the questionnaire must be completed on the site to ensure that the questions are answered without influences or distractions.
- Reassure the subject that he/she has sufficient time for completing the questionnaire on the site.

Q: "I don't want bother filling out this questionnaire."

- Remind the subject that all subjects must complete this questionnaire as part of the study procedures and that the data are important to assess the impact on visual impairment on subjects' lives.
- Assure the subject that it does not take much time.
- EQ-5D-5L is optional for visits 5, 7 and 9, early termination and unscheduled visit. Offer the subject not to complete the EQ-5D-5L questionnaire for those visits but encourage the subject to complete the VILL questionnaire at all visits.

Q: "I've already filled out this questionnaire."

- Explain that the questionnaires are repeated to identify the changes over time.
- Reassure the subject that there is no need to remember previous answer
- Explain that the current answers have to reflect the current status quo and can differ from previous answers.

Q: "Is this really anonymous?"

- Respond "Yes" and explain that the data are labelled with a subject's identification code only, exactly like the other study data.
- Explain that the responses are not reported separately, but as a group.

Q: “I don’t understand this question.”

- Repeat the question aloud verbatim.
- In case of VILL questions, refer to the VILL concept elaboration sheet (see next section) and provide the subject with the information provided in the VILL concept elaboration.
- Do not interpret the question or explanation of the VILL concept elaboration.
- Remind the subject that there are no right or wrong answers and encourage the subject to answer to the best of his/her ability.

Q: “This question doesn’t apply to me.”

- Explain that there is a reason for including the same questions for all subjects and that the questions are based on interviews with and feedback from other subjects.
- Remind the subject that there are no right or wrong answers and encourage the subject to provide the most relevant answer.
- Stress the importance to complete all questions as part of the study procedures and that the data are important to assess the impact on visual impairment on subjects’ lives.
- Do not force the completion if the subject still has concerns.

Q: “I don’t like any of the answer choices provided.”

- Explain that there is a reason for including these answer choices and that there are the same answer choices for all subjects.
- Remind the subject that there are no right or wrong answers and encourage the subject to provide the most relevant answer.

Q: “How do you think I did?”

- Remind the subject that there are no right or wrong answers.
- Explain that you are not trained to interpret individual results.

5.1 VILL concept elaboration

Should the subject actively enquire about the meaning of a certain question of the VILL, the study staff can provide the information given in the Concept Elaboration Sheet. No additional information and no own interpretations of the questions should be provided by site staff. Ultimately, the subjects should be reminded that there are no right or wrong answers and questions should be answered to the best of the subject’s ability.

6 Acknowledgement

The MACUSTAR consortium receives funding from the Innovative Medicines Initiative 2 Joint Undertaking under grant agreement No 116076. This Joint Undertaking receives support from the European Union’s Horizon 2020 research and innovation programme and EFPIA.

More information about the MACUSTAR project will be available shortly on www.macustar.eu.

More information on IMI is available on www.imi.europa.eu.

Disclaimer: The content of this document reflects the authors’ view. Neither IMI nor the European Union or EFPIA are responsible for any use that may be made of the information contained herein.
