# Supplementary material for: Clinical study protocol for a low-interventional study in intermediate age-related macular degeneration developing novel clinical endpoints for interventional clinical trials with a regulatory and patient access intention—MACUSTAR
Source: Trials. 2020 Jul 18;21:659. doi: 10.1186/s13063-020-04595-6 (PMC7368769; doi:10.1186/s13063-020-04595-6)
Supplement: Supplementary file 2 — Additional file 2. Procedure Manual. Retinal Imaging [file 13063_2020_4595_MOESM2_ESM.pdf]

Procedure Manual

Retinal Imaging

MACUSTAR Study

ECR-AMD-2017-13

Version 1.1

1 Certification of Imaging Systems and Photographers/ Technicians, and Application for User Accounts

Each system used in the course of the study as well as each photographer/technician will need to be certified by the GRADE Reading Center **prior** to acquisition of study data.

GRADE will certify imaging systems and photographers/technicians separately.

Certification requires the following steps:

1. Check whether the systems at the clinical site fulfill the requirements for certification
2. Fill in and send the application forms below to the GRADE Reading Center:
   a. Application for certification of imaging systems
   b. Application for submitter user account to access the GRADE Internet Portal
   c. Application for registration/certification of photographers/technicians
3. Upon notification of registration from GRADE Reading Center the photographers/technicians may upload images, separately for each modality, for certification
4. GRADE Reading Center will confirm certification of imaging systems and photographers/technicians. A certificate will be issued for the photographers/technicians and sent to the respective technician to be archived in the MACUSTAR Investigator File.

1.1 System Requirements for Imaging Systems

1.1.1 Spectralis platform

A Spectralis system (Manufacturer Heidelberg Engineering) is required that allows for all of the four different categories:

1. At least cSLO blue (BR) and near-infrared (NIR) reflectance imaging, preferable also green reflectance imaging (the latter to be used for Multicolor (MC) imaging)
2. At least cSLO blue autofluorescence (BAF), preferable also green autofluorescence (GAF)
3. SD-OCT imaging
4. Fluorescein angiography (FA)

If other Spectralis devices which are able to perform SD-OCT (e.g., Spectralis OCT, Spectralis OCT^{Plus}) are available at the site, these devices can be used for OCT imaging.

Spectralis systems in combination with HRA2 (only cSLO) can be accepted as well. In this case, please contact GRADE for further clarification.

Spectralis Software Version 5.6 or newer is required. If your clinical site does not have at least version 5.6 of the software, please contact your local representative of Heidelberg Engineering. If you have not all modalities listed above, the required hardware upgrade might be easily feasible. In this case, contact Heidelberg Engineering as well.

1.1.2 Cirrus SD-OCT imaging

Different Zeiss OCT systems are used in the study. Please check which system is implemented at your site.

The following different Zeiss OCT imaging systems are accepted:

Zeiss Cirrus HD-OCT 400/4000

Zeiss Cirrus HD-OCT 500/5000

Zeiss Cirrus Photo 600/800

1.1.3 Standard colour fundus photography (CFP)

- Standard fundus camera
- Digital
- Angle of view: 30°-40°
- Minimum resolution for color images: 1536 x 1536 pixels
- Capability to export images as single files in DICOM format[1]. (**Please note that PNG, BMP, TIF, or JPG format are not acceptable)**

The Zeiss Visucam, followed by any other Zeiss platform is strongly recommended. Generated false-color images based on multispectral (also called "Multicolor", "Pseudocolor" or "Optos") SLO imaging are not acceptable for standard colour fundus photography.

1.1.4 Quantitative autofluorescence (qAF)

For qAF imaging a Spectralis system (manufacturer Heidelberg Engineering) with quantitative autofluorescence imaging mode including installation of the qAF reference standard and upgrade to HEYEX 6.0 software is required.

---

[1] In contrast to previous project, we require DICOM format for CFP. The ancillary information saved in this format is considered important for the development of automated analysis algorithm within the project.

1.1.5 OCT-Angiography (OCT-A)

While no standard platform for OCT-A is defined; the following list ranks the preferred systems. If you have more than one of these platforms available at your site, please use the one with the highest rank on the list.

1. Zeiss Cirrus HD-OCT 5000 with AngioPlex module (preferred)
2. Zeiss PLEX Elite 9000 SS-OCT
3. Heidelberg Engineering Spectralis OCT-2

In case you have a different device for OCT-Angiography available at your site (e.g. Optovue Angio OCT, Topcon OCT A (DRI OCT Triton), please contact the GRADE Reading Center for confirmation if the device can be used for MACUSTAR study and further instructions.

1.1.6 Swept-source optical coherence tomography (SS-OCT)

For SS-OCT imaging, the following OCT device is accepted only.

- Zeiss PLEX Elite 9000 SS-OCT

1.2 Application for Certification of Imaging Systems

Each imaging system needs to be certified by the GRADE Reading Center before its application in the study.

1.3 Application for Submitter User Accounts to Access the GRADE Internet Portal

In order to upload study data to the GRADE Reading Center via the GRADE Internet Portal, submitter user account(s) must be requested.

1.4 Application for Registration/Certification of Photographers and Technicians

Each photographer/technician must be registered at the GRADE Reading Center.

1.5 Certification of Photographers/Technicians

Upon receiving feedback from the GRADE Reading Center (via e-mail) about the status of the certification of systems, assignment of submitter user accounts, and successful

registration of photographers/technicians, the next step is the certification of the registered photographers/technicians.

The aim of certification is the confirmation of:

- Availability of adequate systems at the clinical site.
- Familiarity of the photographer/technician with the imaging assessment protocol.
- Successful data export and upload of the image data to the GRADE Internet Portal.

Grandfathering: Photographers/Technicians who were previously certified by the GRADE Reading Center, can apply for grandfathering for the respective modalities and imaging platforms. Prerequisites are:

- Individual certification and/or submission of images within another study according to protocol ("completely acceptable") must have taken place within the past 12 months.
- The imaging system the photographer/technician was certified for has to be of the same type as the imaging system used for the MACUSTAR study.

If photographers/technicians would like to apply for grandfathering, they must send a request to GRADE (see appendix **Fehler! Verweisquelle konnte nicht gefunden werden.**).

To become certified photographers/technicians have to submit image data of the following standard modalities/platforms:

- Spectralis imaging (including confocal scanning laser ophthalmology (cSLO) autofluorescence (FAF), spectral-domain optical coherence tomography (SD-OCT)) Certification for FA is waived, currently not required.
- Cirrus SD-OCT imaging (optical coherence tomography (OCT))
- Standard colour fundus photography (CFP, including red-free images)

To become certified photographers/technicians have to submit image data of the following innovative imaging modalities:

- Quantitative autofluorescence (qAF)
- OCT-Angiography (OCT-A)
- Swept-source optical coherence tomography (SS-OCT)

Please note:

- At each clinical site, there must be at least one photographers/technicians certified per modality prior to beginning recruitment of subjects. It is recommended to have at least two photographers/technicians for each modality for backup.
- If a certified photographer leaves a site, the site should give a notice to GRADE. Then, GRADE will deactivate the account of the respective photographer and a new photographer should be appointed.

For certification, photographers/technicians have to acquire images of **two eyes with retinal pathology** per modality; the eyes for certification do not have to show intermediate age-related macular degeneration.

- The two test eyes should but not have to be from one test subject per modality/platform.
- You do not need to use the identical “test eyes” for all modalities/platforms, i.e., you may submit images from different subjects at the discretion of the clinical site among different modalities/platforms in order to gain certification status.

A successful certification requires the submission of a **complete data set** for each modality/platform. This comprises the following steps:

1. **Image acquisition/assessment of images** according to the respective imaging protocol.
2. **Data export** of acquired images/test results of the respective systems.
3. **Labeling of folders and files** exported in step 2 according to the guidelines.
4. **Generation of a ZIP compressed archive**.
5. **Upload of the ZIP compressed archive to the GRADE Internet Portal**.

Following submission, the GRADE Reading Center will carefully review the data, particularly with respect to completeness and quality.

For each submission, GRADE will provide feedback through the GRADE Internet Portal, including detailed advice in case of incomplete or inadequate submissions.

Additionally, GRADE will notify the respective photographer/technician about his/her certification status via e-mail. A certificate will be issued and sent to the photographer/technician to be archived to the MACUSTAR Investigator File.

In case of failure, GRADE will request a resubmission of certification images from the respective photographers/technicians.

Based on previous experience and with the aim to reduce the number of non-acceptable certification requests, please find below the most common reasons for non-acceptance in the past.

Table 1: Most common reasons for non-acceptance of certification requests.

**Reason for non-acceptance**	**Problem resolution**
Wrong image labeling	Ensure to label all files and folders
Missing bitmap file for verification purpose	For Spectralis imaging, ensure to always export an additional bitmap file of the field 2 images when submitting data in the Heidelberg raw data format (e2e) as described on the flash cards.
Incomplete set of images	Ensure to submit a complete set of images, e.g. for Zeiss OCT imaging always submit 2 macular cube scans per eye or for FA imaging always submit 3 early phase images of the study eye.
Wrong settings used	Ensure to use the settings, e.g. for Spectralis imaging take care to activate the ART* mode, EDI** mode if required and to use the correct resolution settings.

*ART mode - Automated Real-Time mode (only applicable to Heidelberg Engineering Spectralis systems). Several individual frames are automatically averaged to improve signal to noise ratio
**Enhanced-Depth-Imaging.

2 Masking and Entering Subject Data

The GRADE Reading Center will accept only data with masked subject identity. Images with personalized data (date of birth, name, etc.) will not be accepted by the GRADE Reading Center.

Because of different modalities and systems/software in the study, we can give only guidelines for the masking and entering of subject data. Please take note of the differences in entering data for certification and for regular study visits, illustrated in this section.

Table 2: Entering masked subject data (Heidelberg Explorer).

Parameter	Content	Example
**Last Name**<br>Certification<br>Study visit	 Study name-Cert-StudyID-SiteID[1]<br>Study name	 MACUSTAR-cert-313-111<br>MACUSTAR
**First Name**<br>Certification<br>Study visit	 Photographer[2]-XX[3]<br>StudyID[4]-SiteID[1]-SubjectID[5]	 Doe_John-01<br>313-111-2222
**Middle Name**	[Leave blank]	Not applicable
**Date of Birth**	01.01.1900	Not applicable (so always use ‘01.01.1900’)
**Gender**	Male	Not applicable (so always use ‘Male’)
**Subject ID**<br>Certification<br>Study visit	 [Leave blank] or free space to enter a number<br>SubjectID[5]	 <br>2222

[1]SiteID – The ID of the clinical site is a 3-digit number, provided by EVICR.net Coordinating Centre (AIBILI)
[2]Photographer – Last name_First name
[3]XX – number of submission, starting at 01
[4]StudyID – fixed study identifier 313
[5]SubjectID – Unique 4-digit number

3 Acquisition of Images

3.1 Checklist before Start

1. Subject ID is available
2. The GRADE Reading Center has registered all imaging systems for all modalities/platforms.
3. The GRADE Reading Center has certified the photographer for the respective modality/platform.
4. Pharmacological pupil dilation performed.

3.2 Field Definitions

The following section contains a short definition of each field (listed in recommended order).

Table 3: Field definitions

Field Name	Explanation
**Fundus Reflex**	Anterior segment, focused on the lens, nasal and temporal canthi at the edge of the image field
**Field 1M (Disc)**	Temporal edge of the disc in the center of the image
**Field 2 (Macula)**	Fovea in the center, temporal part of the disc at the edge of the image
**Field 3M**	Temporal part of the macula together with the center of the fovea with approximately one disc diameter to the edge of the image

Depending on special pathology of the respective study subject, additional images may be acquired at the discretion of the clinical site.

4 Spectralis imaging (including cSLO autofluorescence, SD-OCT and FA)

General settings for Spectralis imaging:

- Mask subject data.
- Capture all images in **high-speed mode** (resolution 768 x 768 pixels) and with the respective angles as described in table 4.

4.1 Combined cSLO+SD-OCT

Important:

1. Combined cSLO+SD-OCT must be performed before fluorescein angiography (i. e. dye application)

2. Do not <u>manually correct</u> the automated segmentation of OCT bands prior to submission of OCT data to the GRADE Reading Center!

Table 4: Spectralis imaging protocol.

Parameter	Setting
Eyes/Visit	Study eye: V1, V3 to V9, Early Termination Visit<br>Fellow eye: V1, V3, V6, V9, Early Termination Visit
Resolution	high-speed mode “Normalized” activated
Follow-up Function (see section below)	Define the screening examination (V1) as reference and use the follow-up function from V4 for all follow-up visits.
**Step 1** - NIR	• Start with the study eye using the NIR mode<br>• Field 2 (Macula) must be centered on the fovea and encompass the temporal part of the optic disc<br>• Focus the image, orienting on the foveal blood vessels<br>• Adjust sensitivity and laser power<br>• Activate the ART* mode selecting at least 30 single frames and press Acquire to obtain a mean infrared image of field 2*<br>• De-activate the ART* mode and switch filter wheel from “A” to “R”
**Step 2** – Multicolor or “red-free” (blue) reflectance	• Switch from NIR to multicolor or “red-free“ (blue) mode[1]<br>• Adjust sensitivity and focus (usually -0,5 diopters)<br>• Activate the ART* mode selecting at least 30 single frames and press **Acquire** to obtain a mean “red-free“ image of field 2*<br>• De-activate the ART* mode<br>• Go to either step 3a OR 3b – depending on the capabilities of your system (step 3a is preferred)

Parameter	Setting
**Step 3a** – combined blue+green FAF– *if not available, go to Step 3b*	• Switch filter wheel from "A" to "S" and change to combined green and blue FAF imaging<br>• Increase and adjust sensitivity for BAF/GAF imaging (be aware of under and overexposure)<br>• Activate the ART* mode selecting at least 30 single frames and press **Acquire** to obtain a mean FAF image of field 2 and field 1M<br>• De-activate the ART mode<br>• Go to step 4<br><br>*Make sure that you have obtained two fields for FAF imaging (2 and 1M)*
**Step 3b** – central blue FAF *only applies if no combined blue+green FAF is possible – see Step 3a*	• Switch filter wheel from "R" to "A" to change from "red-free" to FA mode for BAF<br>• Increase and adjust sensitivity for blue FAF imaging (be aware of under and overexposure)<br>• Activate the ART* mode selecting at least 30 single frames and press **Acquire** to obtain a mean FAF image of field 2 and field 1M<br>• De-activate the ART* mode<br><br>*Make sure that you have obtained two fields for FAF imaging (2 and 1M)*
**Step 6 –** horizontal central SD-OCT B-scan (OCT protocol 1)	• Switch to NIR+SD-OCT<br>• **Acquire** one single horizontal B-scan, centered on the fovea, ART* mode with 30 single frames
**Step 7** - Volume scan 1 (fast) (OCT protocol 2)	• **Acquire** one raster scan of 20° x 20°, centered on the fovea, consisting of 25 B-scans (distance 240 µm), ART* mode, each B-scan consists of 4 single frames
**Step 8** – Volume scan 2 (dense + EDI**) (OCT protocol 3)	• **Acquire** one raster scan of 30° x 25°, EDI mode**, centered on the fovea, consisting of 241 B-scans (distance 30 µm), ART* mode, each B-scan consists of 9 single frames
**Repeat all eight steps for the fellow eye at V1 (screening), V3, V6, V9 (final visit) and Early Exit Termination Visit**	

*ART mode - Automated Real-Time mode (only applicable to Heidelberg Engineering Spectralis systems). Several individual frames are automatically averaged to improve signal to noise ratio.
**Enhanced-Depth-Imaging.
[1] If available (preferred), perform multicolor imaging. If your device does not allow for multicolor imaging, acquire alternatively "red-free" reflectance images only. Please note that "red-free" may be called "blue" reflectance with your device (which is considered to be identical for the study).

Activate the respective protocol in the **NIR + OCT** mode and adjust settings according to table 4. Press the **ART button**, and then start imaging by clicking **Acquire**.

Follow-up examinations

The follow-up function is only used for study visits, starting from V4 onwards (Month 6). Starting at V4 (Month 6), set the images of the screening visit (V1) as the reference and all subsequent visits (V4, V5, V6, V7, V8, V9 and VEE) are imaged according to this reference.

For V3 (validation visit), do not use the follow-up function, i. e. do not use V1 images as the reference.

The **follow-up function** of the Heidelberg Eye Explorer ensures that respective scans are captured at the same retinal location. Thus, define the screening (V1) examination of each of the scan protocols from V4 onwards as reference in the image overview of each subject – prior to acquisition of follow-up scans.

Open the context menu by right-clicking **Progression > Set Reference**. This will mark the selected image as reference image.

Now you can initiate follow-up examinations by clicking the **Follow-Up** button in the image capturing control panel.

4.2 Fluorescein angiography (FA)

Fluorescein angiography (FA) is performed in the usual manner - intravenous injection of 5 ml of a 5% or 10% fluorescein solution with an injection time from start to finish of **5 seconds**.

FA imaging must be performed with your Heidelberg Spectralis imaging system (standard for the current study) you must not use a fundus camera for FA.

The following section summarizes the acquisition parameters for FA.

Table 5: FA imaging protocol.

Parameter	Setting
Eyes/Visit	FA is not mandatory. It can be performed at the discretion of the investigator at each visit. In this case, images are to be transferred to the reading center.
Settings	Standard lens with a field of 30°x30°, high-speed mode, “Normalized” activated, ART mode (15 frames).

Time	Phase	Study eye	n	Fellow eye	n
0 s	Start of injection	Field 2	1		
5 s	End of injection	Field 2	1		
< 60 s	Early phase	Field 2	3		
1min – 3min	Middle phase	Fields 1M, 2 (ART* mode)	2	Fields 1M, 2 (ART* mode)	2
5 min	Late phase	Field 2 (ART* mode)	2	Field 2 (ART* mode)	1

10 min	Late phase	Field 2 (ART* mode)	1	Field 2 (ART* mode)	1
		**Total number of images**	**10**		**4**

* ART mode - Automated Real-Time mode (only applicable to Heidelberg Engineering Spectralis systems). Several individual frames are automatically averaged to improve signal to noise ratio.

4.3 Data Export

For Spectralis imaging submit a total of 4 cSLO images (NIR, MC or at least BR (field 2), combined blue and green FAF (BAF/GAF) or at least blue FAF (BAF) (fields 2 and 1M) per eye. For Spectralis OCT imaging, submit three OCT scans per eye. Additionally, if FA imaging was performed at the discretion of the investigator, submit 14 individual FA images (of both eyes) to the GRADE Reading Center.

If you have captured multiple images of a required field/time, prepare **only one version** (the one with the best quality) for data export.

Export image data from Heidelberg Explorer as follows:

1. Create an export folder per modality (e.g. OCT, cSLO, FA).
2. Label the export folder and all exported image files exactly.
3. Export all images of both eyes per modality (OCT, cSLO and FA) into one E2E file.
4. For verification purpose, additionally export a single bitmap image (PNG, BMP, or TIF) of the horizontal central OCT B-scan (Protocol 1), the field 2 blue and green (or blue) FAF image, and the field 2 FA image (middle phase) per eye.
5. Export the files into the export folder or move or copy the files into this folder after export.

Follow the instruction below to export images from Heidelberg Explorer (Heyex) :

1. Move icons of all frames of both eyes per modality from a visit that is requested by protocol into the lightbox. Mark all images in the lightbox and right click the marked images. In the context menu click on **Export > as E2E.**
2. Move the required images for bitmap export of both eyes into the lightbox. Mark and right click both images. In the context menu click on **Export > as Picture Fehler! Verweisquelle konnte nicht gefunden werden.**.
3. After clicking an export option the respective export dialog window opens. In these dialog windows check boxes and enter data according to the examples. Do **not** check

“Anonymize data” on the E2E file export dialog window, otherwise the Heidelberg Explorer will remove even the masked subject data from the exported E2E file.

4. Compress the export folder into a ZIP archive. Upload the ZIP file to the GRADE portal.

5 Cirrus SD-OCT imaging

General settings:

- Enter the pseudonymized subject data.
- Signal strength of the scan should be at least 6/10.
- Follow the protocol given in table 6.

Important: Do not manually correct the automated segmentation of OCT bands prior to submission of OCT data to the GRADE Reading Center!

Table 6: SD OCT scan protocol.

**Parameter**	**Setting**
Eyes/Visit	Study eye: V1, V3 to V9, Early Exit Termination Visit<br>Fellow eye: V1, V3, V6, V9, Early Exit Termination Visit
Follow-up Function (“Prior scan option”) (see section below)	Define the screening examination (V1) as reference and use the follow-up function from V4 for all follow-up visits. Out of the two macular cube scans at screening, select the better one (at your discretion) as reference for further follow-up visits and always use this scan for reference for all subsequent follow-up visits from V4 onwards.
**Step 1** – Macular Cube (OCT protocol 4)	Macular Cube 200 x 200 **(2 times at each visit)**<br>Signal strength at least 6/10
**Step 2** – HD 21 Line (OCT protocol 5)	HD 21 Line<br>Signal strength at least 6/10
**Repeat all steps for the fellow eye at V1 (screening), V3, V6, V9 (final visit) and Early Termination Visit**	

5.1 Scan Procedure

1. Open the **Scan Acquisition** screen by clicking on **Acquire**.
2. Select **Macular Cube 200 x 200**.
3. Start scan by clicking on **Capture**.
    - The value for signal strength should be at least **6/10**.
    - Ensure centring on the fovea centre.
    - Check for motion artefacts.
4. When scan quality is acceptable click **Save** to save the image.
5. In case of insufficient quality, click **Try Again** followed by **Optimize** before starting another try.

6. Repeat steps 2 to 4 to acquire a second Macular Cube 200 x 200 scan.
7. Select **HD 21 Line**.
8. Start scan by clicking on **Capture**.
9. The value for signal strength should be at least **6/10**.
10. Ensure centring on the fovea centre.
11. Check for motion artefacts.
12. When scan quality is acceptable click **Save** to save the image.
13. In case of insufficient quality, click **Try Again** followed by **Optimize** before starting another try.
14. Click **Finish** to exit acquisition mode.

5.1.1 Follow-up examinations:

The prior scan option is only used for study visits, starting from V4 onwards (Month 6).

Starting at V4 (Month 6), set the images of the screening visit (V1) as the reference and all subsequent visits (V4, V5, V6, V7, V8, V9 and VEE) are imaged according to this reference.

For V3 (validation visit), do not use the follow-up function, i. e. do not use V1 images as the reference.

The **prior scan** option of the Zeiss Cirrus is used for comparison of the current scan to a previous scan of the same subject. Thus, select the screening (V1) examination of each of the scan protocols from V4 onwards as reference in the image overview of each subject – prior to acquisition of follow-up scans. Out of the two macular cube scans at screening, select the better one (at your discretion) as reference for further follow-up visits and always use this scan for reference for all subsequent follow-up visits from V4 onwards.

To activate the **prior scan** option, proceed as follows:

1. Select the Fundus Viewport, just below the Iris Viewport.
2. To compare the current scan to a previous scan of the same patient, select the **prior scan** option above the fundus image.
3. The **Repeat Scan** dialog will be shown.

4. Select the respective V1 scan (Macular Scan 200 x 200 or HD 21 Line) of the same patient and press **OK**. (This will return you back to the **Acquire screen**.)
5. The fundus image is overlaid by the scan pattern for the selected scan type. A small green cross indicates the location of the fixation target.
6. Select **Capture**. The on-screen progress bar indicates the progression of the data acquisition.
7. Proceed to **Review screen**
8. In case of insufficient quality, repeat the steps described above to repeat the acquisition.
9. Click **Finish** to exit acquisition mode.

5.2 Data Export

Follow these steps for data export:

1. Create an export folder (if not done already).
2. Label the export folder.
3. Select **Records > Export Exams** in the menu bar.
4. In the **Patient Export tab**, select the patient to be exported.
5. In the **Exam Export** tab, select visit and eye(s) to export.
6. Uncheck Omit patient Identifiers.
7. Click Browse and select the export folder as destination.
8. Click Export.
9. When the export is successful, a message is displayed; confirm by clicking **OK**.

After export the export folder should at least contain the following subfolders and files:

- A subfolder “data files”
- A file “export.xml”
- A file “zdbdir.ib”

  Do not rename these files.

Before you submit an export folder compress it into a ZIP archive. Upload the ZIP file to the GRADE portal.

6 Standard colour fundus photography (CFP)

6.1 Imaging Protocol

For CFP, two different protocols – the full – protocol (see Table 7) and the minimal protocol (see Table 8) – are used in the study. The latter is defined to save time and burden to patients and study personnel at follow-up visits. At screening, the final visit and early termination visit, the full-protocol must be performed. The following section summarizes the acquisition parameters for CFP.

Table 7: CFP full imaging protocol.

**Parameter**	**Setting**			
Eyes/Visit	Study eye: V1,V3,V9, Early Termination Visit<br>Fellow eye: V1, V3, V9, Early Termination Visit			
Settings	30° to 40° degree angle of view, minimal resolution 1536 x 1536 pixels			
**Mode**	**Study eye**	**n**	**Fellow eye**	**n**
Color	Fundus reflex<br>(Mono)	1	Fundus reflex<br>(Mono)	1
Color	Fields 1M, 2, 3M<br>(Mono)	3	Fields 1M, 2, 3M<br>(Mono)	3
Red free	Field 2<br>(Mono)	1	Field 2<br>(Mono)	1
**Total number of images:**		**5**		**5**

Table 8: CFP minimal imaging protocol.

Parameter	Setting			
Eyes/Visit	Study eye: V4 to V8<br>Fellow eye: V6			
Settings	30° to 40° degree angle of view, minimal resolution 1536 x 1536 pixels			
**Mode**	**Study eye**	**n**	**Fellow eye**	**n**
Color	Field 2<br>(Mono)	1	Field 2<br>(Mono)	1
Red free	Field 2<br>(Mono)	1	Field 2<br>(Mono)	1
**Total number of images:**		**2**		**2**

7 Quantitative autofluorescence (qAF)[2]

Quantitative autofluorescence (qAF) is a new retinal imaging technology that allows for the quantitative measurements of autofluorescence signal intensity at specified retinal areas. Prior to acquisition of qAF images, the hardware and software of your Spectralis imaging device must have been updated.

The first steps for acquisition of qAF imaging are identical to any other well-known cSLO modality in the Heidelberg Eye Explorer, including the opening of the subject and starting the acquisition mode.

Table 9: Quantitative fundus autofluorescence protocol.

Parameter	Setting
Eyes/Visit	Study eye: V2 to V9, Early Termination Visit<br>Fellow eye: V2, V6, V9, Early Termination Visit
Settings	Frame size: 30°x30°, high-speed mode<br>**Please note**: You have to acquire a mean series of 12 frames.
Images	IR: Fundus reflex images of both eyes (anterior segment)<br>qAF: Two movies for both eyes each, centered on the fovea, acquire a mean series of 12 frames per movie, **do NOT compute the mean image when prompted at the end of the acquisition**.

[2]This chapter is based on the imaging protocol “How to acquire quantifiable AF images, Department of Ophthalmology, Columbia University, August 5th, 2013”.

Image Acquisition

15. Capture the fundus reflex IR image of the study eye and also for the fellow eye if applicable to document pupil status.
16. Adjust camera to the posterior pole by using IR-mode to field 2M of the study eye, followed by fine adjustment of the focus to blue light using RF-mode
17. The following qAF acquisition procedures require good subject cooperation and constant eye opening. Consider application of artificial eye drops to improve corneal surface or if the subject seems to blink frequently.
18. Choose **More** and then **qAF** on the touch panel.
19. The reference target appears on top of the live image. Check the reference target. If defects (scratches) or even transillluminating vessels are seen notify the vendor (Heidelberg Engineering) as well as the reading center. Nevertheless, qAF image acquisition should be performed. Depending on the extent of reference damage the qAF images might still be suitable for further analysis.
20. Ensure good positioning of the image frame on the macula (field 2): The temporal half of the optic disc must be seen. The macular area must be in the center of the image. Both vessel arcades (inferior & superior) should be seen.
21. **It is paramount to ensure perfectly even illumination of the image frame as good as possible:**
    a. The image should be **<u>completely illuminated</u>**. **Exclude any shadowing** due to misalignment of the camera or incorrect camera-cornea-distance, especially circular shadowing (multiple dark image corners), dark edges and eyelid shadowing (shadowing of the inferior image half). In case of shadowing re-adjust alignment. Shadowing due to vitreous floaters or anterior segment opacities (lens opacities) should be documented and alerted to the reading center.
    b. Even small differences in gray levels can result in substantial distortions in the color-coded qAF view. Unfortunately, the color-coded qAF view is not available to the photographer at the time of image acquisition. Therefore, greatest attention must be given to ensure even illumination based on gray level intensities in the live image (much more than in standard FAF image acquisition) throughout the whole acquisition process: Always ensure that upper half and lower half of the image as well as the right and left half of the image show the same amount of illumination. Re-adjust camera alignment as needed.

22. Ensure correct sensitivity setting (or “gain”) by rotating the black wheel on the touch panel in the way that the live image is as bright as possible, but the reference target is not turning red. Overexposure of either reference target or image will lead the reference target to show red. Practically, the sensitivity should be 90, but may be reduced from there, if the reference target turns red.
23. Optimize focus setting. Concentrate on the vessels in the macular area. These should be displayed as sharp as possible.
24. **Perform photo-bleaching for at least 20 seconds** by keeping the camera in position.
    a. Instruct the subject to keep eyes widely open and to avoid eye movements.
    b. Make sure the scanning light beam (blue laser spot) is centered on the center of the pupil
    c. Compensate for any subject movement, to ensure that the complete field 2 is consistently exposed to the blue laser for at least 20 seconds.
25. Capture a field 2 qAF image set after the photobleaching is completed. Make sure the “Mean of 12 fr” on the touch panel is activated. Press “Acquire” on the touch panel to capture a series of 12 images over 12 seconds. During the image acquisition, constantly check the camera’s alignment with the subject. In instances of obvious misalignment or eye movements, immediately acquire another qAF image series.
26. Reset and then capture the second field 2 qAF image set. Have the subject sit back, and pull the camera back to reset all orientation-related settings. After having the subject rest for a few seconds, have the subject return to the chinrest, re-optimize the instrument position/settings (including sensitivity), and acquire a second set of qAF images from the same eye.
    Generally, if time permits, it is advisable to acquire more than two series to have “backup” image sets available in case evaluation at the Reading Center would require the transmission of better images.
27. Repeat steps 12 and 13 for the fellow eye if required.
28. Select “Exit” on the touch panel
29. Press “No” on the following window, exit the acquisition mode and save the images.

7.1.1 Validation

Before the subject leaves the camera, check the qAF images from all datasets for quality (open movie by double clicking on the series). Move scrollbar quickly back and forth to assure:

- Non flickering images

- Images are of equal brightness
- No shadowing is visible
- Each movie of 12 frames should have at least 9 frames that meet these quality criteria in order to be accepted. A complete submission contains two movies per eye.
- Repeat image acquisition if indicated.
- If multiple series have been acquired, initially only the best two series should be sent to the Reading Center.

8 OCT-Angiography (OCT-A)

Table 10: OCT-A scan protocol.

Parameter	Setting
Eyes/Visit	Study eye: V2 to V9, Early Termination Visit<br>Fellow eye: V2, V6, V9, Early Termination Visit
Prior scan/Follow-up Function (see sections below)	Define the examination (V2) as reference and use the follow-up function for all follow-up visits. For details see the respective prior scan or follow-up function section of the device you are using.<br>For Zeiss devices, Out of the two cube scans at baseline (V2), select the better one (at your discretion) as reference for further follow-up visits and always use this scan for reference for all subsequent follow-up visits from V4 onwards.
Repeat the examination for the fellow eye at V2, V6, V9 (final visit) and Early Termination Visit	
If none of the three listed OCT-A device is available at your site, please contact the GRADE Reading Center	
**PREFERRED OCT-A SYSTEM**	
Zeiss Cirrus HD-OCT 5000 with AngioPlex module	AngioPlex 3 x 3 mm and 6 x 6 mm cube scans **(each two times)**<br>Signal strength 8 or more
**ALTERNATIVE OCT-A SYSTEMS**	
Zeiss PLEX Elite 9000 SS-OCT	AngioPlex 3 x 3 mm and 6 x 6 mm cube scans **(each two times)**<br>Signal strength 8 or more
Heidelberg Engineering Spectralis OCT-2	Raster scan of 20° x 20°, centered on the fovea, ART* 7 mode (512 B-scan with 512 A scans), high speed mode<br>Raster scan of 10° x 10°, centered on the fovea, ART* 7 mode (512 B-scan with 512 A scans), high resolution mode

*ART mode - Automated Real-Time mode (only applicable to Heidelberg Engineering Spectralis systems). Several individual frames are automatically averaged to improve signal to noise ratio.

8.1.1 Zeiss Imaging Systems (Zeiss Cirrus HD-OCT 5000 with AngioPlex module and Zeiss PLEX Elite 9000 SS-OCT

General settings:

- Enter the pseudonymized subject data
- Signal strength of the scan should be at least 8/10.
- Follow the protocol given in Table

Important: Do not manually correct the automated segmentation of OCT bands prior to submission of OCT data to the GRADE Reading Center!

8.1.2 Zeiss Cirrus HD-OCT 5000 with AngioPlex module

8.1.2.1 Scan procedure

1. Ensure that the subject is fully prepared for the scan.
2. Select **AQUIRE**. The screen will change to indicate the scan types available.
3. Select **AngioScan 3 x 3**
4. Ask the subject to hold their gaze and head steady. Select **Auto Focus** in the Fundus Viewport.
5. Center the scan beam trough the pupil by left-click the pupil center of the live image in the Iris Viewport.
6. After Auto Focus, check the Iris Viewport to ensure that the pupil is still centered.

   If the Fundus Viewport turns dark, center the pupil, click 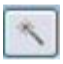, then click the **Auto B/C** button. If additional brightness and contrast changes are necessary, use the appropriate slider controls.
7. Adjust the chinrest manually, if necessary, by using the circular X-Y control. Use Z-control to reach proper working dinstance.
8. Use **Enhance** (polarization) and **Center** (Z-offset) buttons and sliders to help improve the image and center it vertically.
9. Adjust the region of the iris visible in the Iris Viewport.
10. Focus the iris image using the controls to the right of the viewport. Primarily use the Z controls for focusing and the mouse scroll wheel for fine adjustments.
11. Center the pupil in the Iris Viewport by clicking the center of the pupil. A centering target overlays the video image for posterior segment scans.
12. Select **Capture**. An on-screen progress bar indicates how the data acquisition is progressing. The time depends on the fixation stability of the eye. If the instrument has difficulty tracking, the progress bar stalls and the user will have the option to either realign the eye or to turn tracking off.
13. Proceed to the **Review screen** where you will determine whether or not the scan is acceptable or if it should be repeated.
14. Repeat steps 2 to 13 to aquire a second AngioPlex Scan 3x3

15. Repeat steps 2 to 13 twice but select AngioPlex Scan 6x6 on the scan acquisition screen to acquire two 6x6 cube scans.
16. Click finish to exit the acquisition mode.

8.1.2.2 Check scan quality

Once data acquisition is complete, scans must be reviewed for acceptance prior to being released for analysis. The **Scan Quality Check Screen** appears automatically, upon completion of data acquisition.

OCT scan should be complete in all windows without missing data. Color density should be the same from end to end. Signal strength should be 6 or greater.

8.1.2.3 Follow up examinations: Repeat Scan

The prior scan option is only used for study visits, starting from V4 onwards (Month 6).

Starting at V4 (Month 6), set the images of the baseline visit (V2) as the reference and all subsequent visits (V4, V5, V6, V7, V8, V9 and Early Termination Visit) are imaged according to this reference.

For V3 (validation visit), do not use the follow-up function, i. e. do not use V2 images as the reference.

The **prior scan** option of the Zeiss Cirrus is used for comparison of the current scan to a previous scan of the same subject. Thus, select the baseline (V2) examination of each of the scan protocols from V4 onwards as reference in the image overview of each subject – prior to acquisition of follow-up scans. Out of the two macular cube scans at baseline, select the better one (at your discretion) as reference for further follow-up visits and always use this scan for reference for all subsequent follow-up visits from V4 onwards.

To activate the **prior scan** option, proceed as follows:

1. Follow the procedure described in 8.1.2.1. Scan procedure, steps 1-13.
2. If you want to compare the current scan to a previous scan of the same subject, select the **prior scan** option (just above the fundus image).
3. This action will bring up the **Repeat Scan** dialog.
4. The Repeat Scan dialog lists all previous scans in the same scan type category for this subject by exam date and eye and shows the fundus image for the scan you select.

5. Select the scan of interest and press **OK**. This action will return you back to the **Acquire screen**. The system will apply all the parameters of the previous scan and displays the fundus image from that exam. (NOTE: The chinrest will move during this adjustment.)
6. If you selected **Repeat Scan**, your settings for the current scan should be correct. Select **Capture**. The progress bar will indicate the data progress
7. Proceed to the Scan quality check screen.

8.1.3 Zeiss PLEX Elite 9000

8.1.3.1 Scan procedure

For details of the scan procedure please see the SS-OCT section

8.1.3.2 Data export

Export the all files into an export folder you previously created.

8.1.4 Heidelberg Engineering Spectralis OCT-2

General settings for OCT-A:

- Mask subject data.
- Capture all images in **high-resolution mode, ART 7** and with the respective angles as described in 10

Important:

- You may perform OCT-A imaging after the Spectralis imaging as described in 'MACUSTAR Procedure Manual – Standard Retinal Imaging' – however, remember to switch to <u>**high-resolution** mode when acquiring the 10° x 10° raster scan.</u>

Important: Do not <u>manually correct</u> the automated segmentation of OCT bands prior to submission of OCT data to the GRADE Reading Center!

Table 11: Spectralis OCT-A imaging protocol.

Parameter	Setting
Eyes/Visit	Study eye: V2 to V9, Early Termination Visit<br>Fellow eye: V2, V6, V9, Early Termination Visit
Resolution	high speed or high-resolution mode
Follow-up Function	Define the baseline examination (V2) as reference and use the follow-up function from V4 for all follow-up visits. Do not use the follow-up function for V3.
**Step 1** – OCTA (HS 20° x 20°)	• Select the OCTA mode<br>• Ensure that the **high-speed** mode is selected<br>• Set the ART* to 7<br>• Set the imaging volume to a raster of 20° x 20° (512 section [B-scans] containing 512 A-scans)<br>• Field 2 (Macula) must be centered on the fovea and encompass the temporal part of the optic disc<br>• Focus the image, orienting on the foveal blood vessels<br>• Adjust sensitivity and laser power<br>**Acquire** one raster scan of 20° x 20°
**Step 2** – OCTA (HR 10° x 10°)	• Select the OCT-A mode<br>• Ensure that the **high-resolution** mode is selected<br>• Set the ART* to 7<br>• Set the imaging volume to a raster of 10° x 10° (512 sections [B-scans] containing 512 A-scans)<br>• Field 2 (Macula) must be centered on the fovea and encompass the temporal part of the optic disc<br>• Focus the image, orienting on the foveal blood vessels<br>• Adjust sensitivity and laser power<br>• **Acquire** one raster scan of 10° x 10°
**Repeat all seven steps for the fellow eye at V2, V6, V9 (final visit) and Early Termination Visit**	

*ART mode - Automated Real-Time mode (only applicable to Heidelberg Engineering Spectralis systems). Several individual frames are automatically averaged to improve signal to noise ratio.

8.1.4.1 Check scan quality

Once data acquisition is complete, scans must be reviewed for acceptance prior to being released for analysis. The **Scan Quality Check Screen** appears automatically, upon completion of data acquisition.

OCT scan should be complete in all windows without missing data.

8.1.4.2 Follow up examinations

The follow-up function is only used for study visits, starting from V4 onwards (Month 6). Starting at V4 (Month 6), set the images of the baseline visit (V2) as the reference and all subsequent visits (V4, V5, V6, V7, V8, V9 and Early Termination Visit) are imaged according to this reference.

For V3 (validation visit), do not use the follow-up function, i. e. do not use V2 images as the reference.

The **follow-up function** of the Heidelberg Eye Explorer ensures that respective scans are captured at the same retinal location. Thus, define the baseline (V2) examination of each of the scan protocols from V4 onwards as reference in the image overview of each subject – prior to acquisition of follow-up scans.

Open the context menu by right-clicking **Progression > Set Reference**. This will mark the selected image as reference image.

Now you can initiate follow-up examinations by clicking the **Follow-Up** button in the image capturing control panel.

9 Swept-source optical coherence tomography (SS-OCT)

Parameter	Setting
Eyes/Visit	Study eye: V2 to V9, Early Termination Visit<br>Fellow eye: V2, V6, V9, Early Termination Visit
Prior scan/Follow-up Function<br>(see sections below)	Define the examination (V2) as reference and use the follow-up function for all follow-up visits. For details see the respective prior scan or follow-up function section of the device you are using.<br>For Zeiss devices, out of the two cube scans at baseline (V2), select the better one (at your discretion) as reference for further follow-up visits and always use this scan for reference for all subsequent follow-up visits from V4 onwards.
Cube scan	**Cube 12 mm x 12 mm (1024 x 1024)**, ensure that the highest resolution is selected (i.e. 1024 x 1024).
**Repeat the examination for the fellow eye at V2, V6, V9 (final visit) and Early Termination Visit**	

9.1.1 Zeiss PLEX Elite 9000

The following steps describe the acquisition of SS-OCT and OCT-A scans using a Zeiss PLEX Elite 9000 device.

9.1.1.1 Scan procedure

1. Ensure that the subject is fully prepared for the scan.
2. **NOTE**: The first scan selection and scan of the day will run slightly slower than all later scans.
3. Select **AQUIRE** to open the scan acquisition screen. The screen will change to indicate the scan types available.
4. Select **AngioPlex Scan 3x3 for OCT-A or Cube 12x12 for SS-OCT imaging**.
5. Ask the subject to hold their gaze and head steady. Select **Auto focus** in the Fundus Viewport.
6. Left click the pupil center of the live image in the Iris Viewport to center the scan beam through the pupil.
7. Select **Auto focus**. Check that the pupil is still centered (Iris Viewport).

- **NOTE**: If the Fundus Viewport turns dark, center the pupil, click, then select **Auto Brightness/Contrast**.

8. To finetune brightness and contrast, select 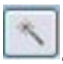. This will open the Fundus Image and OCT Tomogram slider controls. See **Fehler! Verweisquelle konnte nicht gefunden werden.**
9. Use the manual chinrest controls to finetune the location of the eye in the Iris Viewport.
10. Use Z controls (left-right arrows or mouse scroll wheel) to reach the proper working distance by bringing the iris image into focus.
11. Use **Enhance** (polarization) and **Center** (**Z-offset**) buttons and sliders to the left help you improve the scan image quality and center it vertically.
12. Center the pupil in the Iris Viewport by clicking the center of the pupil. A centering target overlays the video image.
13. Direct your attention to the Fundus Viewport, just below the Iris Viewport. (If you will not be using prior scan data for comparison, skip to step 16.)
14. Select **Capture**. The on-screen progress bar indicates how the data acquisition is progressing. The time depends on the fixation stability of the eye. If tracking is on, and the progress bar stalls, you will have to either realign the eye or turn tracking off.
15. Proceed to Scan Quality Check screen (see below).
16. Repeat steps 3 to 15 to aquire a second AngioPlex Scan 3x3 for OCT-A imaging
17. Repeat steps 3 to 15 twice but select AngioPlex Scan 6x6 on the scan acquisition screen to acquire two 6x6 cube scans for OCT-A imaging.
18. Repeat steps 3 to 15 to acquire a Cube Scan 12x12 (1024 x1024) for SS-OCT imaging.
19. Click finish to exit the acquisition mode.

9.1.1.2 Check Scan Quality

Once data acquisition is complete, scans must be reviewed for acceptance prior to being released for analysis. The Scan Quality Check Screen appears automatically, upon completion of data acquisition.

1. Check that the focus is sharp and clear, preferably with good visibility of the branching blood vessels.
2. Check that the scan overlay in the Fundus image is centered on the fovea, and that it is illuminated uniformly and without dark corners.
3. Ensure that the Fundus image is illuminated uniformly, and without dark corners.

4. Ensure that there few to no artifacts that may cast shadows on the OCT scan.
5. Check for saccades. En Face image should have minimal saccades and no saccades through the area of interest. A saccade can be detected by discontinuities in the appearance of blood vessels.
6. Make sure that the scan is complete in all windows without missing data.
7. Ensure color density is same from end to end.
8. Check the signal strength. It should be 8 or higher.

9.1.1.3 Follow-up examinations

The prior scan option is only used for study visits, starting from V4 onwards (Month 6).

Starting at V4 (Month 6), set the images of the baseline visit (V2) as the reference and all subsequent visits (V4, V5, V6, V7, V8, V9 and Early Termination Visit) are imaged according to this reference.

For V3 (validation visit), do not use the follow-up function, i. e. do not use V2 images as the reference.

The **prior scan** option of the Zeiss Cirrus is used for comparison of the current scan to a previous scan of the same subject. Thus, select the baseline (V2) examination of each of the scan protocols from V4 onwards as reference in the image overview of each subject – prior to acquisition of follow-up scans. Out of the two macular cube scans at baseline, select the better one (at your discretion) as reference for further follow-up visits and always use this scan for reference for all subsequent follow-up visits from V4 onwards.

To activate the **prior scan** option, proceed as follows:

1. Follow the procedure described in chapter 9.1.1, steps 1-13.
2. To compare the current scan to the previous scan of the same subject, select the **Prior Scan** option. This action will bring up the Repeat Scan dialog which will list all previous scans in the same scan type category for this subject (by exam date and eye) and shows the fundus image for the scan you select.
3. Select the scan of interest and select **OK**. (You will return to the Acquire screen)
4. If you selected **Repeat Scan**, the settings for the current scan should be correct.
5. Select **Capture**. The progress bar will indicate the data progress.
6. Proceed to Scan Quality Check screen.

9.1.2 Data export

Export the all files into an export folder you previously created.

10 Data Export

Submit a total of **10 individual images for V1, V3, V6, V9 and Early Termination Visit, 4 individual images for V6** and **2 individual images for V4, V5, V7, V8, resp.,** for color fundus photography.

If you have captured multiple images of a required field, prepare **only one version** (the one with the best quality) for data export.

- Export each image as a separate file from the respective imaging system in **DICOM format** into an export folder you previously created
- (**Please note that PNG, BMP, TIF or JPG formats are not acceptable)**

Label the export folder and all exported image files exactly as described previously.

Before you submit the export folder to GRADE compress it into a ZIP archive. Upload the ZIP file to the GRADE portal.

11 Acknowledgement

The MACUSTAR consortium receives funding from the Innovative Medicines Initiatives 2 Joint Undertaking under grant agreement No 116076. This Joint Undertaking receives support from the European Union’s Horizon 2020 research and innovation programme and EFPIA.

More information about the MACUSTAR project will be available shortly on www.macustar.eu.

More information on IMI is available on www.imi.europa.eu.

Disclaimer: The content of this document reflects the authors’ view. Neither IMI not the European Union or EFPIA are responsible for any use that may be made of the information contained herein.
