# Supplementary material for: Clinical study protocol for a low-interventional study in intermediate age-related macular degeneration developing novel clinical endpoints for interventional clinical trials with a regulatory and patient access intention—MACUSTAR
Source: Trials. 2020 Jul 18;21:659. doi: 10.1186/s13063-020-04595-6 (PMC7368769; doi:10.1186/s13063-020-04595-6)
Supplement: Supplementary file 3 — Additional file 3. Model consent form. [file 13063_2020_4595_MOESM3_ESM.docx]

| MODEL CONSENT FORM  FOR AMD SUBJECTS |
| --- |
| **CLINICAL STUDY TITLE:**  Development of novel clinical endpoints for interventional clinical trials with a regulatory and patient access intention in patients with intermediate age-related macular degeneration (AMD) - MACUSTAR |
|  |

1. I declare that I read the Information Sheet and this form and that I voluntarily accept to participate in the clinical study.
2. I understand that my consent is the legal basis for processing of my personal data and samples in the manner and for the purposes described in the Information Sheet.
3. I have been fully informed of the nature, purpose, risks and likely duration of the study, as well as what is expected from me.
4. I have had the opportunity to ask questions about all aspects of the study and I have understood the answers and the information which has been given to me. I had enough time to decide.
5. At any time I can ask the study physician for complementary information. I will receive during the whole clinical study and whenever I wish, information about its progress. Any new relevant information that could modify my consent will be given to me by the study physician.
6. I agree to follow all the instructions given to me during the clinical study. I agree to entirely cooperate with the study physician and to inform him/her immediately of all changes in my health or well-being of any nature, and of all unexpected or unusual symptoms of any type which may occur.
7. I have understood that I am free to withdraw from the clinical study at any time, without having to justify my decision and without compromising in any way the quality of my medical care. I have understood that the study physician and the Sponsor has the right to withdraw me from the study and will inform me about the reason.

**I consent to the processing of my personal data in the manner and for the purposes described in the Information Sheet/Informed Consent Form.**

I will receive one copy of the Information Sheet and Informed Consent Form. The original remains in the ***clinical site.***

***Name of Participant (printed letters): _______________________________________________***

***Signature: ___________________________________________ Date: _______/_____/____***

I confirm that the information in the Information Sheet/ Informed Form Consent were explained accurately, and apparently understood by the participant and that consent was freely given by the study participant.

***Name of Impartial Witness or Legally Acceptable Representative (if applicable):***

***______________________________________________________________________________***

***Signature: ___________________________________________ Date: _______/_____/____***

I confirm that I have explained to the above mentioned participant nature, purpose, and potential risks of the above mentioned clinical study.

***Name of Investigator (printed letters):______________________________________________***

***Signature: ___________________________________________ Date: ______/_____/____***

**MODEL CONSENT FORM**

**FOR AMD SUBJECTS**

**For the collection of blood samples for genetics in the scope of MACUSTAR clinical study**

1. I agree with the collection of blood samples for genetics in the scope of the MACUSTAR clinical study.
2. I agree that the blood samples collected will be in a coded form.
3. I agree to the storage and analysis of my blood samples for genetic purposes in the context of the MACUSTAR clinical study.
4. I consent in the shipping to and storage of the samples at the study Sponsor’s facilities, at University Hospital of Bonn, in Bonn, Germany.
5. I consent in the shipping of my blood samples from the Sponsor facilities to the Radboud University Medical Centre in Nijmegen, in The Netherlands.
6. I authorize the processing and genetic analysis of my blood samples.
7. I am aware that the samples will be destroyed after being analyzed.
8. I have read the Information Sheet and have had the opportunity to ask questions. I was informed that my participation in the MACUSTAR clinical study is not affected by this optional blood collection and subsequent genetic analysis. I can still participate in this clinical study if I decline the collection, storage and analysis of any blood samples for genetic purposes.
9. I have been informed that I can withdraw my consent given at any time without giving reasons. In case of withdrawal and based on my decision the remaining biomaterials and collected data will either be destroyed/ deleted or anonymized for further use.

**I consent to the collection of blood samples for genetics in the manner and for the purposes described in the Information Sheet/Informed Consent Form.**

I will receive one copy of the Information Sheet and Informed Consent Form. The original remains in the ***clinical site.***

***Name of Participant (printed letters):___________________________________________***

***Signature: ___________________________________________ Date: _______/_____/____***

I confirm that the information in the Information Sheet/ Informed Form Consent were explained accurately, and apparently understood by the participant and that consent was freely given by the study participant.

***Name of Impartial Witness or Legally Acceptable Representative (if applicable):***

***______________________________________________________________________________***

***Signature: ___________________________________________ Date: _______/_____/____***

I confirm that I have explained to the above mentioned participant the nature, purpose, and potential risks of the above mentioned clinical study.

***Name of Investigator (printed letters):________________________________________________***

***Signature: ___________________________________________ Date: ______/_____/____***

**MODEL CONSENT FORM**

**FOR AMD SUBJECTS**

**For the collection of blood samples for biobanking in the scope of MACUSTAR clinical study**

1. I agree with the collection of blood samples for biobanking in the scope of the MACUSTAR clinical study.
2. I agree that the blood samples collected will be coded form.
3. I agree to the storage of my blood samples for biobanking purposes in the context of the MACUSTAR clinical study.
4. I consent in the shipping to and storage of the samples at the study Sponsor’s facilities, at University Hospital of Bonn, in Bonn, Germany.
5. I consent in the shipping of my blood samples from the Sponsor facilities to the Radboud University Medical Centre in Nijmegen, in The Netherlands.
6. I consent in the use of my blood samples for measurements of genetic factors and biomarkers at institutes or companies inside or outside the MACUSTAR Consortium. These measurements will only be done following a positive decision by the MACUSTAR Consortium and an Ethics Committee.
7. I authorize the processing of my blood samples for the purposes described in the Information Sheet/Informed Consent Form.
8. I have read the Information Sheet and have had the opportunity to ask questions. I was informed that my participation in the MACUSTAR clinical study is not affected by this optional blood collection. I can still participate in this clinical study if I decline the collection, storage and analysis of any blood samples for genetic and biomarker purposes.
9. I have been informed that I can withdraw my consent at any time without giving reasons. In case of withdrawal and based on my decision the remaining biomaterials and collected data will either be destroyed/ deleted or anonymized for further use.

**I consent to the collection of blood samples for biobanking in the manner and for the purposes described in the Information Sheet/Informed Consent Form.**

I will receive one copy of the Information Sheet and Informed Consent Form. The original remains in the ***clinical site.***

***Name of Participant (printed letters):_______________________________________________***

***Signature: ___________________________________________ Date: _______/_____/____***

I confirm that the information in the Information Sheet/ Informed Form Consent were explained accurately, and apparently understood by the participant and that consent was freely given by the study participant.

***Name of Impartial Witness or Legally Acceptable Representative (if applicable):***

***______________________________________________________________________________***

***Signature: ___________________________________________ Date: _______/_____/____***

I confirm that I have explained to the above mentioned participant the nature, purpose, and potential risks of the above mentioned clinical study.

***Name of Investigator (printed letters):________________________________________________***

***Signature: ___________________________________________ Date: ______/_____/____***
